# Supplementary material for: A promoter interaction map for cardiovascular disease genetics
Source: eLife. 2018 Jul 10;7:e35788. doi: 10.7554/eLife.35788 (PMC6053306; doi:10.7554/eLife.35788)
Supplement: Transparent reporting form [file elife-35788-transrepform.docx]

***eLife’s* transparent reporting form**

We encourage authors to provide detailed information *within their submission* to facilitate the interpretation and replication of experiments. Authors can upload supporting documentation to indicate the use of appropriate reporting guidelines for health-related research (see [EQUATOR Network](http://www.equator-network.org/%20)), life science research (see the [BioSharing Information Resource](https://biosharing.org/)), or the [ARRIVE guidelines](http://www.plosbiology.org/article/info:doi/10.1371/journal.pbio.1000412) for reporting work involving animal research. Where applicable, authors should refer to any relevant reporting standards documents in this form.

If you have any questions, please consult our Journal Policies and/or contact us: [editorial@elifesciences.org](mailto:editorial@elifesciences.org).

**Sample-size estimation**

- You should state whether an appropriate sample size was computed when the study was being designed
- You should state the statistical method of sample size computation and any required assumptions
- If no explicit power analysis was used, you should describe how you decided what sample (replicate) size (number) to use

Please outline where this information can be found within the submission (e.g., sections or figure legends), or explain why this information doesn’t apply to your submission:

We used a sample size of three in our study. Due to the high cost of sequencing for Hi-C techniques, higher sample sizes become prohibitive. We note that three replicates is higher than the generally used two replicates. We discuss the reproducibility of our data in the Results and Methods section, and provide quantification in Figure 1 – Figure Supplements 2 and 3.

**Replicates**

- You should report how often each experiment was performed
- You should include a definition of biological versus technical replication
- The data obtained should be provided and sufficient information should be provided to indicate the number of independent biological and/or technical replicates
- If you encountered any outliers, you should describe how these were handled
- Criteria for exclusion/inclusion of data should be clearly stated
- High-throughput sequence data should be uploaded before submission, with a private link for reviewers provided (these are available from both GEO and ArrayExpress)

Please outline where this information can be found within the submission (e.g., sections or figure legends), or explain why this information doesn’t apply to your submission:

The iPSC-CM differentiations were performed successively, three independent times. Each new differentiation represents a different aliquot of the same iPSC line, which was generated from one individual. Therefore, we consider each differentiation to represent a soft biological replicate.

The PCHi-C and RNA-seq data are derived from these three replicates. Detailed information on the filtering steps used in all downstream analyses of these data are provided in the Methods.

**Statistical reporting**

- Statistical analysis methods should be described and justified
- Raw data should be presented in figures whenever informative to do so (typically when N per group is less than 10)
- For each experiment, you should identify the statistical tests used, exact values of N, definitions of center, methods of multiple test correction, and dispersion and precision measures (e.g., mean, median, SD, SEM, confidence intervals; and, for the major substantive results, a measure of effect size (e.g., Pearson's r, Cohen's d)
- Report exact p-values wherever possible alongside the summary statistics and 95% confidence intervals. These should be reported for all key questions and not only when the p-value is less than 0.05.

Please outline where this information can be found within the submission (e.g., sections or figure legends), or explain why this information doesn’t apply to your submission:

All statistical tests and associated p-values are reported in the main text when first discussed (i.e. Results), as well as in the figure legend of the accompanying figure. We detail the analysis design for relevant figures (e.g. for calculating enrichment of promoter interactions overlapping certain features) in the Methods section.

(For large datasets, or papers with a very large number of statistical tests, you may upload a single table file with tests, Ns, etc., with reference to sections in the manuscript.)

**Group allocation**

- Indicate how samples were allocated into experimental groups (in the case of clinical studies, please specify allocation to treatment method); if randomization was used, please also state if restricted randomization was applied
- Indicate if masking was used during group allocation, data collection and/or data analysis

Please outline where this information can be found within the submission (e.g., sections or figure legends), or explain why this information doesn’t apply to your submission:

Not applicable

**Additional data files (“source data”)**

- We encourage you to upload relevant additional data files, such as numerical data that are represented as a graph in a figure, or as a summary table
- Where provided, these should be in the most useful format, and they can be uploaded as “Source data” files linked to a main figure or table
- Include model definition files including the full list of parameters used
- Include code used for data analysis (e.g., R, MatLab)
- Avoid stating that data files are “available upon request”

Please indicate the figures or tables for which source data files have been provided:

Most analyses and all figures involve the promoter interaction data. We have deposited the raw read files as well as the CHiCAGO output files and processed interactios files at ArrayExpress (E-MTAB-6014). We have also deposited RNA-seq data at ArrayExpress (E-MTAB-6013). We also include the significant interaction files used for all analyses in the paper as supplementary file attachments.

We are not providing code directly, as we believe that the Methods section details all analyses sufficiently to reproduce our results using the source files we provide. These details include the program name and version used for each analysis, along with parameters.
